# Chromosomal Inversion Associated With Diet Differences in Common Quails Sharing Wintering Grounds

**DOI:** 10.1002/ece3.71792

**Published:** 2025-08-20

**Authors:** Celia Vinagre‐Izquierdo, Ines Sanchez‐Donoso, Jennifer A. Leonard, José Domingo Rodríguez‐Teijeiro, Carles Vilà

**Affiliations:** ^1^ Conservation and Evolutionary Genetics Group Estación Biológica de Doñana (EBD‐CSIC) Seville Spain; ^2^ Institut de Recerca de la Biodiversitat (IRBio) Universitat de Barcelona (UB) Barcelona Spain; ^3^ Departament de Biologia Evolutiva, Ecologia i Ciències Ambientals Universitat de Barcelona Barcelona Spain

**Keywords:** chromosomal inversion, diet, genotyping by sequencing, migration, stable isotopes

## Abstract

Chromosomal inversions can contribute to genetic differentiation and ecological adaptation. In common quails ( *Coturnix coturnix* ), a large chromosomal inversion encompassing over 1200 genes is associated with key phenotypic traits, including increased body size, darker throat pigmentation, and reduced flight efficiency, which may influence migratory behavior. We hypothesized that the presence of resident common quails in the south of the Iberian Peninsula is the consequence of the high frequency of this chromosomal inversion, found in a high proportion of the breeding individuals in the region. We surveyed one wintering population in southern Spain and analyzed the genomic composition, morphology, and deuterium, nitrogen, and carbon stable isotope composition of primary feathers. Our results revealed the coexistence of birds with different karyotypes and morphologies that also differed in migratory behavior, as inferred from the comparison of the stable isotope signature in feathers. While quails with the inversion showed limited evidence of migratory movements, quails without the inversion seemed to have reached the area from other latitudes. Interestingly, our results also revealed that these migratory quails that reached this population in winter had differences in their diet. Thus, two separately evolving chromosomal lineages, characterized by the presence/absence of the inversion, coexist in the wintering area, leading to differences in morphology, behavior, and resource use. Due to the lack of recombination in the inversion, the divergence is expected to continue increasing.

## Graphical abstract

A large chromosomal inversion in common quails ( *Coturnix coturnix* ) is associated with phenotypic traits. In a wintering population in southern Spain, we found all three karyotypes coexisting but showing differences in migratory behavior and diet, as exemplified by the isotopic signature. This suggests ecological divergence driven by the inversion.

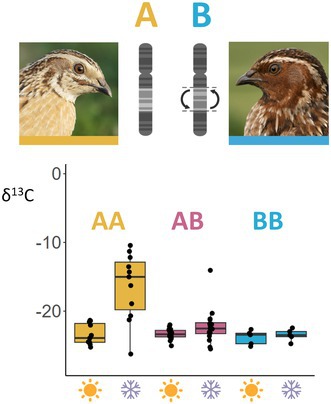

## RESUMEN

Las inversiones cromosómicas pueden contribuir a la diferenciación genética y a la adaptación ecológica. En las codornices comunes ( *Coturnix coturnix* ), una gran inversión cromosómica que abarca más de 1.200 genes está asociada a rasgos fenotípicos clave, como un mayor tamaño corporal, una pigmentación más oscura en la garganta y una menor eficiencia de vuelo, lo que podría influir en el comportamiento migratorio. Nuestra hipótesis es que la presencia de codornices comunes residentes en el sur de la Península Ibérica es consecuencia de la alta frecuencia de esta inversión cromosómica, presente en una elevada proporción de los individuos reproductores de la región. Estudiamos una población invernante en el sur de España y analizamos la composición genómica, la morfología y la composición de isótopos estables de deuterio, nitrógeno y carbono en las plumas primarias. Nuestros resultados revelaron la coexistencia de aves con distintos cariotipos y morfologías, que además diferían en su comportamiento migratorio, según se pudo inferir a partir de la comparación de la firma isotópica estable en las plumas. Mientras que las codornices con la inversión mostraron escasas evidencias de movimientos migratorios, las codornices sin la inversión parecían haber llegado a la zona desde otras latitudes. Nuestros resultados también mostraron que estas codornices migratorias que alcanzaron esta población en invierno presentaban diferencias en su dieta. Así, dos linajes cromosómicos que evolucionan de forma separada, caracterizados por la presencia o ausencia de la inversión, coexisten en la zona de invernada, lo que da lugar a diferencias en la morfología, el comportamiento y el uso de los recursos. Debido a la ausencia de recombinación en la región invertida, se espera que la divergencia continúe aumentando.

## 1 Introduction

Chromosomal inversions are structural variants characterized by a reversed orientation of a chromosomal segment within its original location (Sturtevant 1921). Multiple genes may be involved in large chromosomal inversions, which can facilitate the accumulation of genetic differences that could affect phenotypes (Thompson and Jiggins 2014; Kirkpatrick 2010; Villoutreix et al. 2020; Guerrero et al. 2012). Consequently, inversions can lead to the evolution of distinct phenotypic syndromes, driving intraspecific and ecological diversification (Wellenreuther and Bernatchez 2018). Chromosomal inversions can be involved in shaping diverse complex phenotypic traits, including social (Avril et al. 2019) and migratory behavior (Tuttle et al. 2016; Lundberg et al. 2017, 2023), or mating strategies (Lamichhaney et al. 2016; Küpper et al. 2016). Inversions contribute to maintaining population polymorphisms (Kirubakaran et al. 2016) and could potentially lead to speciation (Feder et al. 2014). The association between chromosomal inversions, local adaptation, and ecotypic differentiation has been explored in various taxa, such as plants (Twyford and Friedman 2015), insects (Christmas et al. 2019), fish (Hohenlohe et al. 2010; Arostegui et al. 2019; Barth et al. 2019; Jamsandekar et al. 2024) and birds (Hooper and Price 2017).

An example of a very large inversion has been found in common quails (*Coturnix coturnix*, order: Galliformes; Sanchez‐Donoso et al. 2022). This species has a broad Palearctic distribution, ranging from northeastern Atlantic archipelagos to Mongolia, and extending southward to southern Africa and India (Johnsgard 1988; Del Hoyo et al. 1994; Gallego et al. 1997; Guyomarc'h et al. 1998; McGowan et al. 2023). Common quails are migratory across much of their range and continue to exhibit extensive movements during the breeding season in search of mating opportunities and following suitable habitats, which are closely tied to the seasonality of crops and temperature fluctuations (Rodríguez‐Teijeiro et al. 1992, 2009; Sardà‐Palomera et al. 2012). This chromosomal inversion, which is located in chromosome 1, encompasses over 1200 genes—about 12% of the genome—and has been linked to significant changes in common quail morphology and behavior (Sanchez‐Donoso et al. 2022; Ravagni et al. 2025). Males with this inversion exhibit a larger body size, darker throat pigmentation, and a more rounded wing shape, which suggests reduced flight efficiency and limited dispersal capacity (Holynski 1965). This is further supported by stable isotope analyses which indicate that these birds migrate shorter distances or may not migrate at all (Sanchez‐Donoso et al. 2022).

Quails with the inversion are geographically limited despite the species' high mobility, probably due to their phenotypical particularities associated with lower flight capacity. They are found in the western part of the species range, particularly in the southern Iberian Peninsula, Morocco, and the Macaronesian archipelagos (Azores, Madeira, Canary Islands, and Cape Verde), where most individuals captured carry the inversion (Sanchez‐Donoso et al. 2022; Ravagni et al. 2024). In these regions, quails with and without the inversion coexist and interbreed, but their movements and behavior remain unclear. It is unknown whether the two non‐recombining haplotypes coexist during winter, when migratory individuals move south, or if differences in morphology and behavior lead to variations in their use of environmental resources.

**FIGURE 1:**
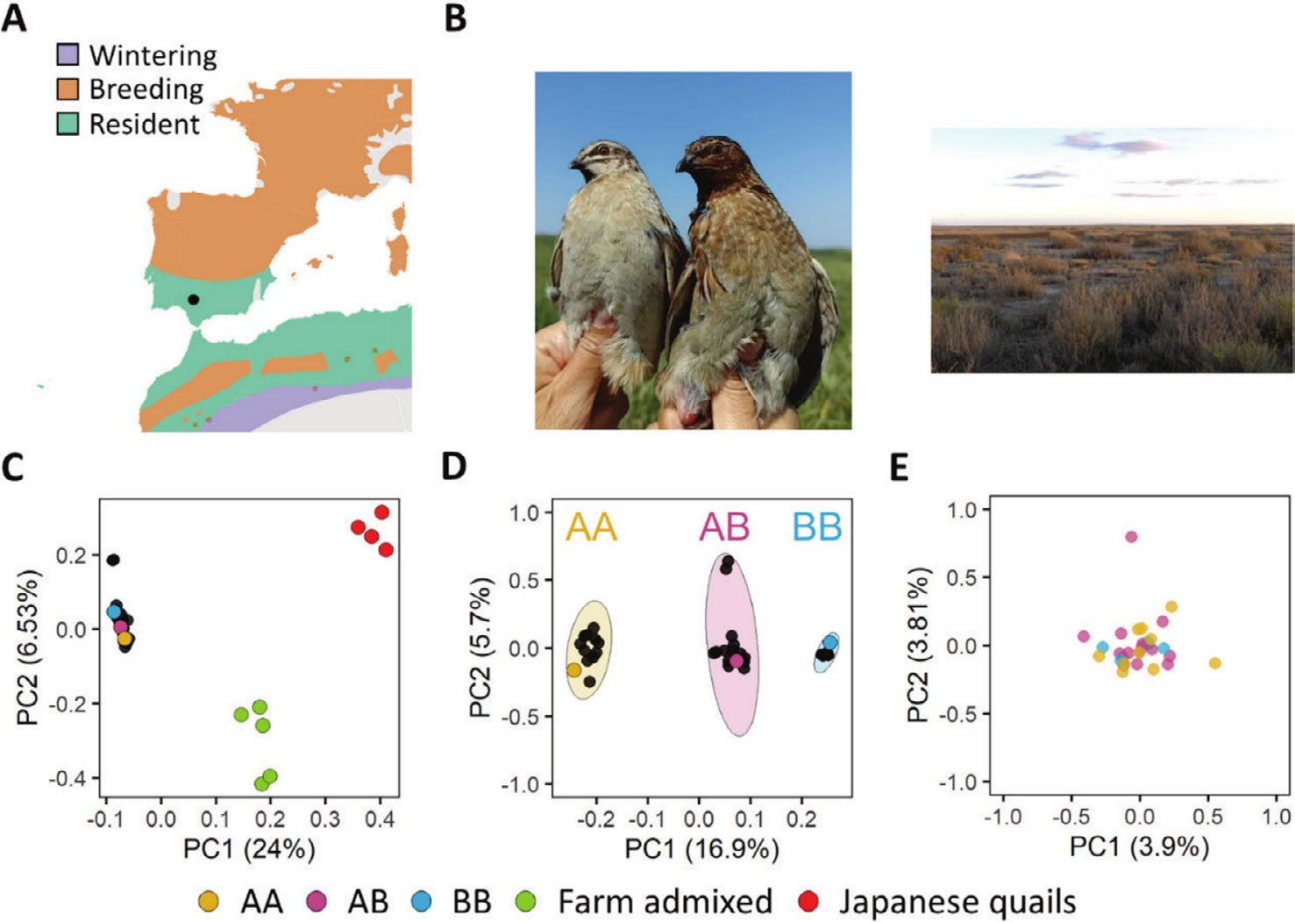
Study area, habitat, and principal component analysis (PCA) of genetic variation in common quails. (A) Location of the study area within the Iberian Peninsula. Colored areas correspond to breeding (orange), resident (green) and wintering areas (purple) for the common quail, according to BirdLife International (2018). Breeding grounds extend to the north reaching Scandinavia and most of the wintering grounds are located in the Sahel, south of the Sahara desert. The study site is within the range where resident quails are expected and it is crossed by migratory quails. (B) Male common quails captured in the same location with (individual with the dark throat) and without the inversion; grasslands in the study area. (C) PCA using the complete set of SNPs (25,416). In red, Japanese quail samples; green, game farm admixed quails (common × Japanese quail); black, wintering quails captured for this study. Yellow, pink and blue mark reference individuals genotyped in a previous study (Sanchez‐Donoso et al. 2022), carrying different inversion karyotypes (inversion haplotype: B; karyotypes: AA, AB and BB) (D) PCA using 3705 SNPs located within the inversion in chromosome 1 and classifying individuals based on their karyotype. (E) PCA using the entire SNP dataset after excluding those in the inverted region and removing one individual identified as first order relative of another one.

Previous research has documented the presence of individuals in the south and west of the Iberian Peninsula and in north‐western Africa during winter, indicating that part of the population could be resident in these regions (Fountoura and Gonçalves 1992; Guyomarc'h 1992; Guyomarc'h and Fontoura 1993; McGowan et al. 2023; Figure 1A). The high overlap between the areas with resident quails and the presence of the inversion suggests that this inversion could explain the resident population. If wintering quails in this part of the distribution corresponded mainly to individuals carrying the inversion, in homozygosis (BB) or heterozygosis (AB), this could suggest spatial segregation in winter among the two chromosomal variants.

This study aimed to investigate whether the presence of wintering quails in the southwest of the Iberian Peninsula could correspond to quails carrying the chromosomal inversion which did not migrate. If this is the case, a genetically unique part of the population (differentiated over ~12% of the genome) would be subject to different selective pressures, potentially driving distinct strategies in resource‐limited winter habitats. We characterized the genomic composition of wintering quails in this region and measured stable isotope composition of primary feathers grown during the winter and the breeding seasons to explore the interplay between genetic composition, migratory behavior, and morphology. We seek to better understand how seasonal migration and genetic diversity shape populations in environments with limited resources, particularly during winter.

## 2 Materials Methods

### 2.1 Field Sampling

Quails were captured in a protected grassland at the northeastern edge of Doñana National Park (37° 03′12.7″N, 6° 18′14.9″W), in the southwest of the Iberian Peninsula (Figure 1A,B). This area experiences moderate winters with variable precipitation between years. We carried out 25 one‐night field campaigns during the winter (November to February) of 2021–2022. We selected these months because preliminary data showed that they correspond to the time period when males captured in the area did not have either swollen cloacas (< 4.5 mm in size), or proctodeal foam, which are potential signs of sexual activity (Guyomarc'h et al. 2001; Seiwert and Adkins‐Regan 1998), nor very large fat deposits that are associated with migration (Saint‐Jalme et al. 1988; Guyomarc'h et al. 1990). Two groups of 10 mist nets, approximately 10 m in length each net, were set up before sunset at two sites about 2.5 km apart. We placed electronic decoys with common quail vocalizations overnight by each set to attract them. We regularly checked the nets during the night to ensure that no birds were injured. At sunrise, we drove quails that had approached overnight towards the nets.

We weighed the captured individuals with a precision scale (±0.5 g). We measured tarsus length, including the intertarsal joint, with a digital vernier caliper (±0.01 mm), as outlined by Sutherland et al. (2004), as well as the width of the pectoral lipid band (viewed through the skin) and the width of the cloacal aperture. We measured the maximum folded wing length using a zero‐stop ruler (±0.5 mm), with the wing flattened and the lateral curvature straightened, and the length of each primary feather and the second secondary from one wing with a pin ruler (±0.5 mm). All measurements were conducted by the same person (IS‐D) to minimize inter‐observer variability. We categorized cheek pigmentation into three categories (light, medium, and dark). We also categorized the beak pigmentation into the same three categories and measured beak height, width (taking as reference the most distal point of the nostrils in both measures) and length (from the extreme of the beak to the most proximal point of the nostril bulge). We sampled two primary feathers, one grown at the birth place and another one at the wintering grounds (following the molting pattern described in Saint‐Jalme and Guyomarc'h 1995), for stable isotope analyses. Feathers were stored dry in paper envelopes. We collected about 100 μL of blood from each quail for genomic analysis and stored it in NAP buffer (Camacho‐Sanchez et al. 2013). Blood samples were kept cool in the field and frozen at −20°C upon arrival to the laboratory. All birds were banded and released at their capture site.

Field work protocols were evaluated and approved by the CSIC (Spanish National Research Council) Ethics Committee (code 1083/2021) and field protocols and captures were authorized by the regional government (Consejería de Agricultura, Ganadería, Pesca y Desarrollo Sostenible of the Junta de Andalucía, 25/06/2021 and 09/02/2021) and the National Park authorities (ref. 2021107300000935/JPCD/IRM/MDCG/mes, 13/10/2021).

### 2.2 Molecular Genetics

Total genomic DNA was extracted from blood samples stored in NAP buffer using the DNeasy Blood & Tissue Kit (Qiagen Inc.) following the manufacturer's protocols. To enhance the DNA yield, two 100 μL elutions were performed following the extraction process. The concentration of the DNA was measured using a Qubit fluorometer and 50–100 ng/μL was sent to an external facility (The Elshire Group Ltd., New Zealand) for Genotyping by Sequencing (GBS) following the protocol of Elshire et al. (2011). This protocol involves sequencing 64 bp DNA fragments near the restriction enzyme EcoT22I cutting sites.

Sex determination of the individuals was based on phenotypic traits. In cases where sex was unclear, it was determined by the amplification of sex‐specific markers. The differentiation between heterogametic females (ZW) and homogametic males (ZZ) was achieved by visualizing PCR products on a 2% agarose gel differing in size for the two sex chromosomes (Fridolfsson and Ellegren 1999).

### 2.3 Genomic Data Analyses

We demultiplexed sequencing reads using Axe (Murray and Borevitz 2018) with the‐m0 flag. Adapters from paired‐end (PE) sequences were removed with Trimmomatic v. 0.39 (Bolger et al. 2014; v. 0.39). Trimmed PE sequences were merged using BBMerge (Bushnell et al. 2017). Sequences longer than 64 bp were retained with Cutadapt (Martin 2011; v. 0.1.2). We assessed the quality of the sequencing data at each step using FASTQC (Andrews 2010; v. 0.12.1) and MultiQC (Ewels et al. 2016). Single nucleotide polymorphisms (SNPs) were identified using the TASSEL‐5‐GBSv2 (Bradbury et al. 2007; v. 5.0) pipeline, which required adding fake barcodes to the beginning of the reads for program compatibility. Sequences were aligned to the Japanese quail genome (*Coturnix japonica*, RefSeq assembly accession no. GCF_001577835.2) using Bowtie2 (Langmead and Salzberg 2012). After mapping, we filtered the data by retaining only biallelic SNPs excluding scaffolds not located within chromosomes, with a minor allele frequency greater than 0.03, missing data less than 25%, and depth between 5 and 100 using VCFTOOLS v.0.1.13 (Danecek et al. 2011).

We compared the captured individuals with previously genotyped domestic Japanese quails and admixed individuals of game farm origin to identify any potential admixture between wild common quails and birds released from game farms for restocking (Sanchez‐Donoso et al. 2012). We did this with a principal component analysis (PCA) using plink v1.90b6.18 (Purcell et al. 2007). We also assessed the relatedness among sampled individuals using KING version 2.3.2 (Manichaikul et al. 2010), estimating pairwise kinship coefficients with the –kinship flag and identifying IBD (identical by descent) segments with the –ibdseg flag. This allowed the removal of one of the individuals in PCA analyses when first‐order relatives were found. To identify the inverted haplotype in chromosome 1 (labeled B as in Sanchez‐Donoso et al. 2022) we compared the GBS genotypes with a PCA. We included in the analysis the genotype of samples that were previously analyzed by Sanchez‐Donoso et al. (2022) and Ravagni et al. (2024) to be used as a reference to define karyotype groups. We identified individuals as homokaryotypes with the inversion (BB) or without (AA), or heterokaryotypes (AB). We used R version 4.4.2 (R Core Team 2024) using RStudio (Posit Team 2024) and the following R packages to plot the results: cowplot v. 1.1.3 (Wilke 2024), ggplot2 (Wickham 2016), ggpubr v. 0.6.0 (Kassambara 2023), jpeg v. 0.1.10 (Urbanek 2022a), magick v. 2.8.5 (Ooms 2021), png v. 0.1.8 (Urbanek 2022b), tidyverse v. 2.0.0 (Wickham et al. 2019).

To assess whether population structure exists independently of the effects of the chromosome 1 inversion and whether it is associated with geographically distinct breeding populations, we analyzed SNPs located outside the inversion region. This approach ensured that any detected population structure would reflect broader genomic differentiation rather than being driven solely by the inversion. We conducted population structure analyses using both Principal Component Analysis (PCA) and ADMIXTURE (Alexander et al. 2009). PCA was performed using genotype data excluding SNPs within the CHR 1 inversion. ADMIXTURE was run for values of *K* = 1 through 6 to explore potential population clusters. For each *K*, we performed 10 replicate runs, each initialized with a unique random seed, and employed the cross‐validation (CV) procedure to evaluate model fit and identify the most likely *K*. The output files were subsequently used for downstream analysis and visualization. To identify the optimal number of genetic clusters and summarize replicate results, we generated a mapping file and analyzed the Q‐matrices using StructureSelector (Li and Liu 2018) and CLUMPAK (Kopelman et al. 2015).

### 2.4 Stable Isotope Analysis

We cut the apical end of the two primary feathers collected from each individual. The comparison of the isotopic signature of feathers grown in breeding and wintering grounds could inform about the relative magnitude of the migratory movements and differences in diet. Feathers capture the isotopic signature of the place where they were formed. Since common quail primary feathers have well‐defined molt patterns (Saint‐Jalme and Guyomarc'h 1995), the comparison of the isotopic signature of feathers grown during winter versus feathers formed during the spring/summer in breeding grounds can inform about the magnitude of the migratory movements. A portion of each feather (0.3 mg) was placed in a silver capsule to be used for δ^2^H analysis, while another portion (0.3 mg) was used for δ^13^C and δ^15^N analyses and placed into tin capsules. Sample analyses were carried out at the stable isotope laboratory (LIE) of the Doñana Biological Station, certified to ISO9001:2015 and ISO14001:2015 quality and environmental management systems.

Isotope measurements for δ^2^H were performed on H_2_ derived from high‐temperature flash pyrolysis at 1450°C using a Flash HT Plus elemental analyzer coupled to a Delta‐V Advantage isotope ratio mass spectrometer via a CONFLO IV interface (Thermo Fisher Scientific, Bremen, Germany). The stable isotope ratio was expressed in the standard δ‐notation (‰) relative to Vienna Standard Mean Ocean Water (VSMOW). The measurement error based on laboratory standards was ±3‰. The standards used for calibration included CBS, KHS (keratin standards provided by Environment Canada), and LIE‐PA2 (Razorbill feathers as an internal standard). The δ^2^H analyses employed the comparative equilibration approach outlined by Wassenaar and Hobson (2003) and used calibrated keratin isotope reference materials to mitigate the effects of H exchange with ambient water vapor.

For δ^13^C and δ^15^N quantification, samples were combusted at 1020°C using a continuous flow isotope‐ratio mass spectrometry system by means of Flash HT Plus elemental analyzer coupled to a Delta‐V Advantage isotope ratio mass spectrometer via a CONFLO IV interface (Thermo Fisher Scientific, Bremen, Germany). The isotopic composition is reported in the conventional delta (δ) per mil notation (‰), relative to Vienna Pee Dee Belemnite (δ^13^C) and atmospheric N_2_ (δ^15^N). The measurement error based on laboratory standards was ±0.1‰ and ±0.2‰ for δ^13^C and δ^15^N, respectively. The standards used were: IAEA‐600 (caffeine, international standard) and internal standards: LIE‐P‐22 (casein), LIE‐BB (whale baleen) and LIE‐PA (feathers of Razorbill). These laboratory standards were previously calibrated with international standards supplied by the International Atomic Energy Agency (IAEA, Vienna).

We performed a Linear Mixed Model (LMM) for each one of the studied isotopes (δ^2^H, δ^13^C, and δ^15^N) with the function lme() from the R nlme package (Pinheiro and Bates 2000; Pinheiro, Bates and R Core Team 2025) to test the effect of karyotype and growing period of the feather on the isotope composition, as well as their interaction, controlled by individual as a random factor. Significance was evaluated with a chi‐square test by using the Anova() function available in the R car package (Fox and Weisberg 2019). Pairwise comparisons between genomic clusters were done by applying the Sidak family‐wise error rate correction to control for multiple testing using the function emmeans() from the emmeans package (Lenth 2020). Homoscedasticity and normality of the residuals from all models were confirmed by visual inspection of the scatterplots.

### 2.5 Analysis of Phenotypic Data

We examined the association between karyotype and several phenotypic traits: weight, tarsus length (indicators of body size), width of the lateral lipid band (fat accumulation that is larger in birds preparing for migration), cheek pigmentation, cloacal aperture width (larger in sexually active birds), beak length, beak height, and beak width (related to food specialization) and beak pigmentation. To assess wing pointedness, we calculated a modified Holynski Index (Fiedler 2005; Holynski 1965; Lockwood et al. 1998) as in Sanchez‐Donoso et al. (2022), which is based on the lengths of the primary feathers, the second secondary feather, and the length of the wing. This index compares the relative length of the feathers in relation to the longest primary and correlates with flight efficiency (Mönkkönen 1995).

We fitted a linear model for each one of the phenotypic response variables by using karyotype as the explanatory variable of interest. We controlled for age and sex by adding them as factors in the models. Since the effect of age was not significant in any of the tests (results not shown), we excluded it from the final models, controlling only for sex. We also excluded all the interactions, since their effect was not significant in any of the models (not shown). Homoscedasticity and normality of the residuals were confirmed in all models by visual inspection of the scatterplots. Significance was evaluated with an *F*‐test using the Anova() function available in the R car package (Fox and Weisberg 2019). Pairwise comparisons between genomic clusters were done with Tukey's posthoc tests (which correct for multiple testing) using the function emmeans() from the emmeans package (Lenth 2020). We tested the effect of karyotype over cheek pigmentation in males and females separately with two Fisher exact tests with the fisher.test() function in R version 4.4.2 (R Core Team 2024) using RStudio (Posit Team 2024).

In addition, we performed a PCA to assess whether karyotype is associated with consistent multivariate trait patterns across individuals. We included all quantitative phenotypic traits (body weight, wing length, tarsus length, lipid band width, modified Holynski index, cloacal aperture width, cheek and beak pigmentation, beak height, width, and length). All traits were standardized prior to analysis. PCA was performed using the FactoMineR v2.11 (Le et al. 2008) and factoextra v1.0.7 (Kassambara and Mundt 2020) packages in R. To interpret trait covariation along major axes, we examined the loadings (variable coordinates) on the first two principal components.

## 3 Results

From November to February we captured 32 quails, including 14 females and 18 males (Table 1). An additional 52 quails were observed or heard in the proximity of the nets but not captured. Genotyping‐by‐sequencing (GBS) generated a total of 531 million reads. After filtering, 48.2 million reads remained for further analysis. GBS data analysis identified 25,416 SNPs distributed across the genome. To ensure that none of the captured quails derived from animals bred in game farms for restocking, usually admixed with domestic Japanese quail (Sanchez‐Donoso et al. 2012), we used a PCA to compare our samples to domestic Japanese quails and admixed game farm quails, and the results revealed that the captured quails did not show evidence of introgression: all wild quails clustered in together with previously genotyped quails and were very different from Japanese quails and admixed game farm quails (Figure 1C). Only two individuals seemed to be first‐order relatives. Given the small number of related individuals, we maintained all samples in the analyses.

**TABLE 1:** Karyotype frequencies across sexes and age groups for captured quails.

Genotype	AA	AB	BB	Total
Total	11	16	5	32
Sex				
Female	5	7	2	14
Male	6	9	3	18
Age				
yearling (< 1 year)	9	14	3	26
adult (> 1 year)	2	2	2	6

A PCA using only the SNPs from the chromosome 1 inversion (3705 SNPs) showed that wild quails clustered in three groups according to the presence/absence of the chromosomal inversion, as confirmed by the comparison with reference samples from previous studies (Sanchez‐Donoso et al. 2022; Ravagni et al. 2024; Figure 1D). We identified 11 individuals that did not have the inversion (two copies of the A chromosome, without the inversion; karyotype AA), 16 individuals carrying one copy of the inversion (AB) and 5 individuals that were homozygous for the inversion (BB). These results indicated that the population of quails wintering in the study area was not composed only of just one chromosomal lineage or karyotype, as we would expect if winter residents were non‐migratory due to carrying two B (resident) inversion alleles. Instead, the winter population was a mixture of all three possible karyotypes.

We used a PCA representation (Figure 1E) and ADMIXTURE analyses (not shown) using SNPs located outside the inversion to evaluate the degree of genetic isolation between quails from the different karyotypes. The results showed that all three groups were completely undifferentiated and suggested, as previously shown by Sanchez‐Donoso et al. (2022), that the three karyotype groups are not genetically isolated and that gene flow between them is very high outside the region of the inversion.

**FIGURE 2:**
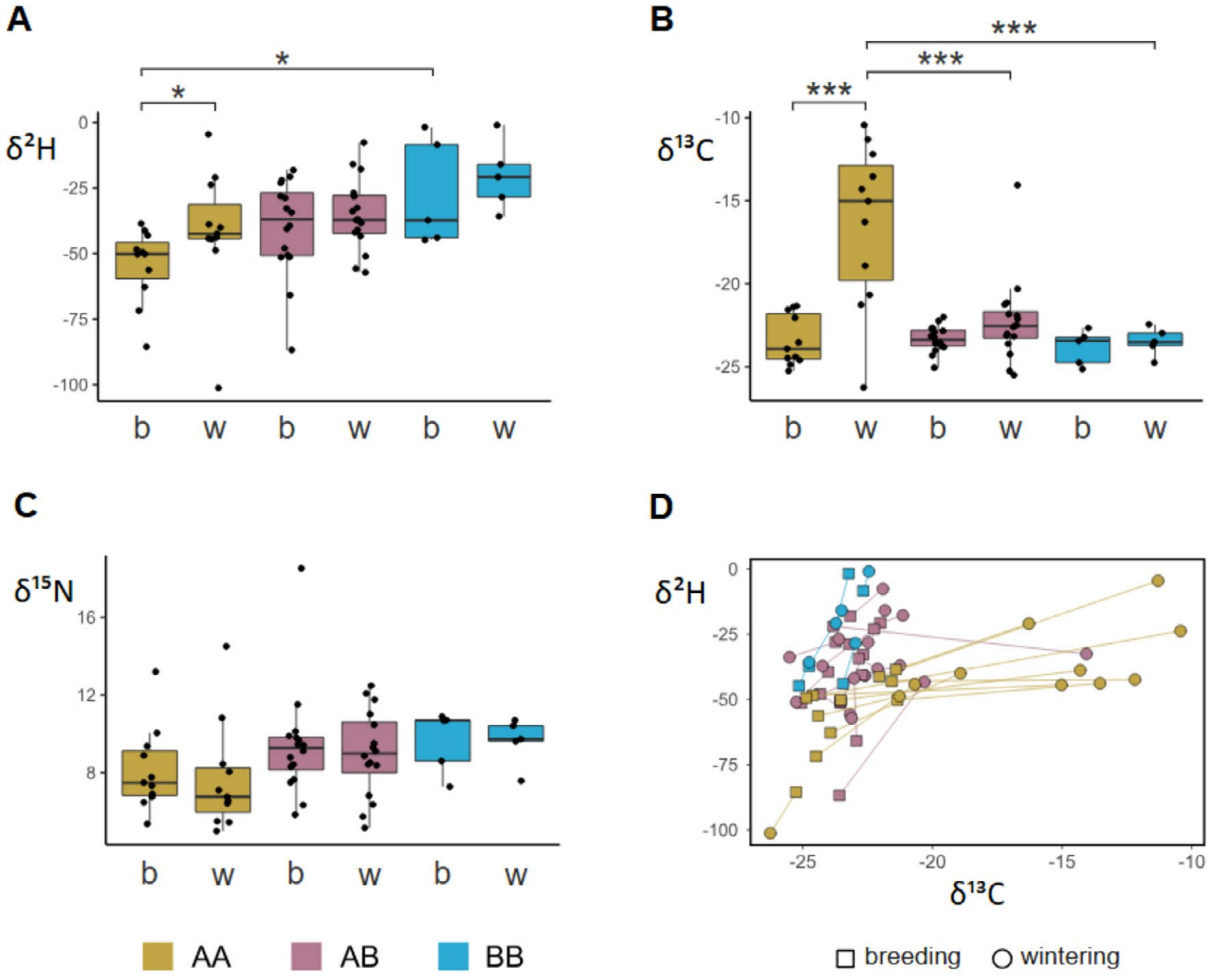
Stable isotopes in two primary feathers from 32 quails captured during winter in one location in southern Spain. One of the feathers was grown during the breeding season when the individual was born (b), the other one was grown during the wintering season when it was captured and sampled (w). Panels (A–C) represent the isotopic values for deuterium (δ^2^H), carbon (δ^13^C) and nitrogen (δ^15^N) for the three karyotypes (AA, AB, BB). Braces mark significant differences (Appendix A; ***p < 0.001; *p < 0.05). Panel (D) represents carbon (δ^13^C) versus deuterium (δ^2^H), linking the feather grown during the breeding season (squares) with the one grown during the wintering season (circles) from the same individual. Wintering feathers of AA quails show similar δ^2^H values to those for AB and BB quails (which suggests that they were formed at similar latitudes) but different δ^13^C values. That suggests AA quails have a different diet during the wintering season while they remain at similar latitudes than the rest of the quails.

To assess if differences in karyotype were associated with differences in migratory and/or feeding behavior, we analyzed the isotopic signature for deuterium (δ^2^H), carbon (δ^13^C) and nitrogen (δ^15^N) of two primary feathers for each individual. Feathers from AA quails grown during the breeding season had significantly different δ^2^H values than those grown in winter. No difference was observed between the feathers of AB and BB quails (Figure 2A; Test 1 from Model H in Appendix A). This result is in agreement with the findings in Sanchez‐Donoso et al. (2022) and suggests that AA quails migrate long distances, while AB and BB quails migrate shorter distances or do not migrate. We observed that AA breeding season feathers showed significantly lower δ^2^H values than the same feathers in BB quails, indicating that AA quails were born or spent the breeding season at different latitudes than BB quails (Figure 2A; see Test 2 from Model H in Appendix A). Results also showed significant differences in δ^13^C values among karyotypes and between seasons, including their interaction (Figure 2B and Model C from Appendix A). We observed big differences in δ^13^C values when comparing breeding and winter feathers for AA quails but no differences when comparing these feathers in AB and BB quails (Test 1 from Model C in Appendix A); δ^13^C values did not differ between karyotypes in feathers grown during the breeding season but were higher in AA than in the other two karyotypes in winter (Test 2 from Model C in Appendix A). These results indicate that the source of C is different for AA quails during winter (see also Figure 2D; Appendix A). We did not find significant differences in δ^15^N neither among karyotypes nor between seasons or their interaction (Figure 2C and Model N in Appendix A). This result indicates that the trophic level was not significantly different between seasons or karyotypes.

**FIGURE 3:**
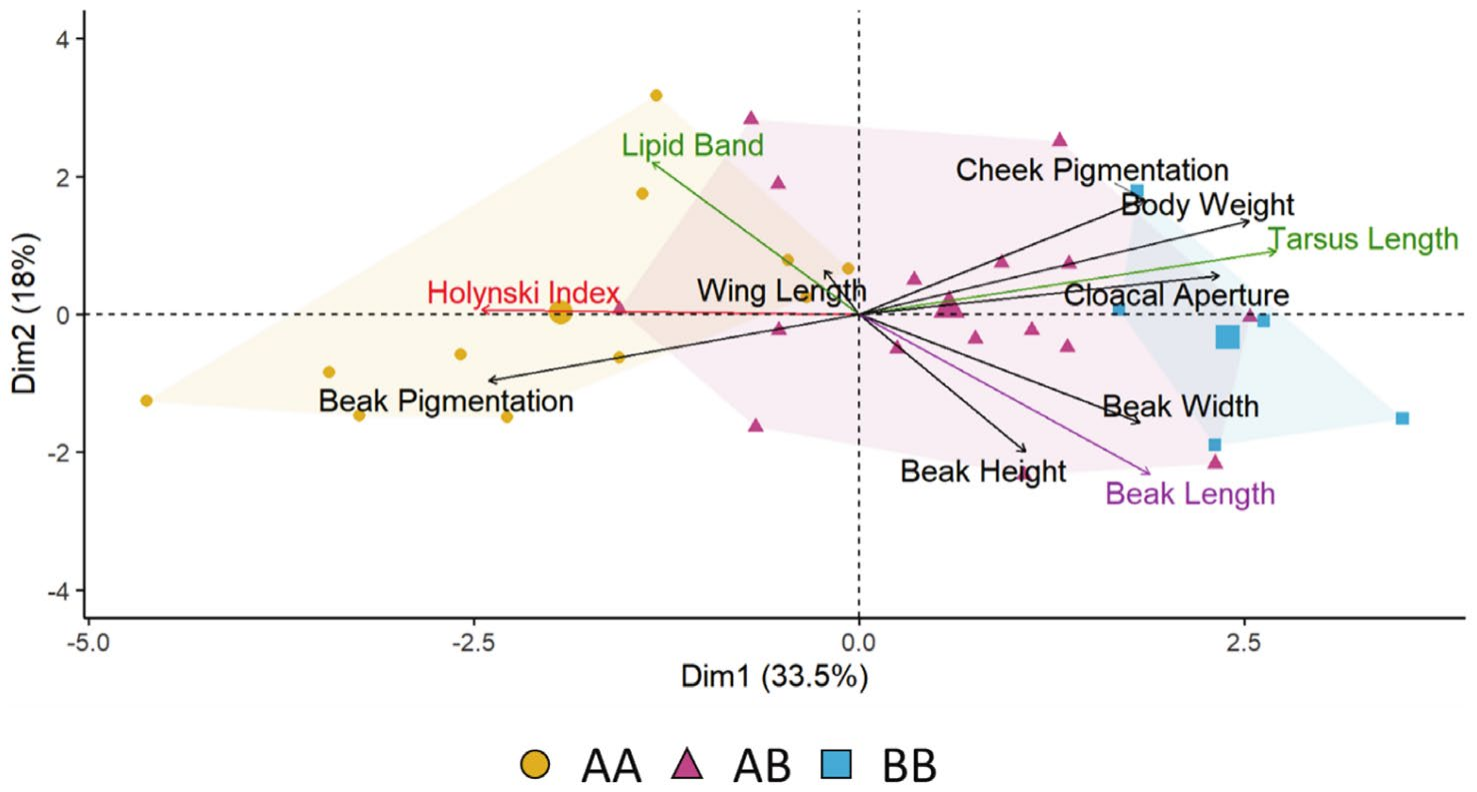
Phenotypic variation by karyotype. PCA of phenotypic data for common quails, colored according to karyotype group (AA: Yellow, AB: Pink and BB: Blue). Arrows point in the direction of the variables and are colored to reflect statistically significant differences between karyotypes: Orange represents variables significantly different across all three karyotypes (AA, AB, and BB); green indicates variables where AA differs significantly from AB and BB; and purple indicates variables with significant differences only between AA and BB.

The comparison of the morphology of quails belonging to the three karyotypes using a PCA revealed significant differences (Figure 3, Appendixes B and C). The first two dimensions accounted for 33.5% and 18.0% of the variance. Key contributors for PC1 included tarsus length (16.54%), weight (14.48%), the modified Holynski index (13.52%) and beak pigmentation (13.06%). This axis separated the three karyotypes. PC2 was primarily influenced by beak length (22.52%), lipid band width (20.49%), beak height (16.65%) and cheek pigmentation (11.75%). Trait loadings (correlations with PC1) indicated covariation among traits. Specifically, lipid band width (*r* = +0.78) and modified Holynski index (*r* = +0.74) both had strong positive loadings on PC1, suggesting that birds with traits typical of migratory preparation (e.g., increased lipid reserves, pointed wings) fall towards the AA cluster. The traits that loaded negatively were tarsus length (*r* = −0.66) and body weight (*r* = −0.70), placing individuals with longer tarsi and larger body mass (BB) on the opposite end. Interestingly, the comparison of beak shape revealed that BB quails had longer beaks (Appendixes B and C). In general, heterokaryotypes (AB) had intermediate morphology for all traits. Males and females differed in weight and in the width of the cloacal aperture (Appendix C), females being heavier and having larger cloacal widths. Other than these, males and females did not differ in the studied phenotypic traits.

## 4 Discussion

Previous research has shown that common quails that have the chromosomal inversion in chromosome 1 may exhibit limited migratory movements (Sanchez‐Donoso et al. 2022). Thus, we hypothesized that the population of quails wintering in the study area in the southwest of the Iberian Peninsula could be mainly composed of quails carrying the inversion (B), in homozygosis (BB) or heterozygosis (AB), that remain in the area throughout the seasons. However, contrary to our expectations, we identified quails without the inversion (AA) together with quails with the inversion (AB and BB) coexisting in the area during winter. The three karyotypes had been identified also among breeding quail males in relatively similar proportions to those we find in winter (Sanchez‐Donoso et al. 2022). Our result thus implies that two very divergent haplotypes (A and B) coexist in the population all year round despite their potential phenotype differences and fitness effects. This raised a critical question: are AA quails wintering in this part of the range a resident population even though they may be typically migratory over most of the species range?

To discern whether the quails are resident or just wintering in the area, we compared stable isotope signatures from feathers. The studied isotopes offer different kinds of information: δ^13^C is dependent on the source of carbon in the diet (C3 vs. C4 plants; O'Brien 2015) and can reveal latitudinal patterns by linking animal movement with climate‐dependent primary productivity (Kelly 2000; Lajtha and Marshall 1994; Hobson 1999; Hobson and Wassenaar 2019); δ^15^N offers insights into the trophic level and habitat conditions, as arid or warmer regions with higher trophic levels generally have elevated δ^15^N values (Hobson and Wassenaar 2019; Layman et al. 2012; Post 2002); δ^2^H is associated with meteoric water and varies geographically due to latitude, elevation, and distance from the ocean (Lajtha and Marshall 1994; Krouse 1989; Poage and Chamberlain 2001; Rubenstein and Hobson 2004). By comparing isotopic proportions in feathers grown during the breeding season with those grown in winter, we can identify whether birds have migrated (Berthold 2003) or have seasonal dietary changes. Quails have a very well defined molt pattern of primary feathers that is interrupted by the fall migration and resumes in the wintering grounds (Heinroth 1927; Glutz von Blotzheim 1973; Cramp and Simmons 1980; Saint‐Jalme and Guyomarc'h 1995). This seasonal molting pattern justifies using feather isotope analysis to track movement between breeding and winter grounds.

If all three karyotypes—AA, AB, and BB—were resident and used the habitat in a similar way throughout the year, they would exhibit similar stable isotope values in feathers formed in wintering and breeding grounds. However, distinct isotope patterns among the karyotypes, particularly for δ^2^H, could suggest differences in migratory behavior. We observed notable differences in δ^13^C and δ^2^H when comparing feathers grown in breeding and winter grounds in AA quails compared to BB and AB quails. This suggests that BB and AB quails in the study area may be residents or may have reduced migratory movements, while AA quails are likely to represent long‐distance migrants that may have arrived from other latitudes. Consequently, the studied area may be home to resident quails, but also to wintering quails and to quails that are present just during the breeding season. It is not clear what can drive some quails to migrate in or out of an area while others are resident, but this does not seem to be a mere result of resource availability.

Interestingly, the observed changes in the isotope signature may also indicate a change in diet. The increase in the proportion of δ^13^C in migratory AA quails in winter implies a change in the source of carbon, with an increase in the contribution of C4 plants (Alisauskas and Hobson 1993; Alisauskas et al. 1998). This implies a change in the diet of these migrant quails after reaching wintering grounds while other quails living in sympatry did not change diet and maintained a similar δ^13^C in summer and winter (Figure 2). This could represent a segregation of trophic resources among coexisting quails when these resources are less abundant. However, this could also be associated with dietary changes linked to the high demands imposed by migratory behavior (Hobson and Clark 1992; Martínez del Rio and Wolf 2005; Wolf et al. 2009). In any case, the timing of this change in diet in AA quails requires further study.

We observed that AA quails exhibited morphological differences compared with quails carrying the inversion (AB and BB), such as weight, tarsus length, wing pointedness, and fat content, and the heterokaryotypes AB tend to have intermediate morphology (Figure 3, Appendixes B and C). These results, based on males and females, are in line with previous studies focused just on males (Sanchez‐Donoso et al. 2022; Ravagni et al. 2024). Interestingly, karyotype groups also differ in beak size, which could further support the idea of dietary divergence, as feeding ecology and beak morphology are closely associated in birds (Gosler 1987; Benkman 1988; Price 1991; Peterson 1993; Barbosa and Moreno 1999; Bardwell et al. 2001; Schondube and Rio 2003; Grant and Grant 2006; Bright et al. 2016; Olsen 2017). This anatomical difference, combined with other phenotypic traits, reinforces the view that AA quails may adopt different feeding and migratory behaviors compared to quails carrying the inversion, with reduced migratory movements.

Our findings complement previous studies in other taxa where genetic polymorphisms underlie variation in migratory strategies, such as in the Atlantic cod ( *Gadus morhua* ), where chromosomal rearrangements are associated with migratory phenotypes (Kirubakaran et al. 2016; Berg et al. 2016; Kess et al. 2019), in pink salmon ( *Oncorhynchus gorbuscha* ), where migratory behavior is associated with population structure (Kovach et al. 2012), and in Eurasian blackcaps ( *Sylvia atricapilla* ), with SNPs linked to migratory distance (Delmore et al. 2020).

In addition to migration, the inversion appears to influence diet, as stable isotope signatures (δ^13^C) indicate that AA individuals have a dietary shift in winter. Many migratory bird species have evolved adaptations that allow them to adjust their diet, enabling them to exploit different food resources encountered along their migratory routes (Bairlein 2002). In the case of the quails, the change in diet occurs in sympatry: while the isotopic signature changes for AA quails in winter, it remains constant for AB and BB quails. Ravagni et al. (2025) found that several genes located within the inversion are involved in lipid metabolism, carbohydrate processing, and protein modification. The changes in these metabolic functions within the inversion support the idea that selection has acted on diet‐related physiology, reinforcing ecological divergence between karyotypes. This supports the hypothesis that diet‐related divergence may be genetically determined, as seen in wolves ( *Canis lupus* ; Pilot et al. 2012), brown boobies ( *Sula leucogaster* ; Jacoby et al. 2023), and killer whales ( *Orcinus orca* ; Reisinger et al. 2016), where genetic differentiation is associated with dietary preferences and habitat use.

As previously seen, AA quails differ morphologically from inversion‐carrying individuals (AB and BB) and we also found differences regarding beak size. Beak size variation supports the hypothesis of divergent foraging niches among karyotypes, given the well‐established link between feeding ecology and beak morphology in birds (e.g., Gosler 1987; Grant and Grant 2006).

These results reveal that the divergence between quails with and without chromosomal inversions extends beyond migratory behavior and phenotypical traits and includes ecological and physiological traits. Thus, the two chromosomal lineages (non‐recombining haplotypes) that coexist in the southwest of the Iberian Peninsula may be subject to very different selective forces and represent diverging evolutionary paths. Our study provides critical insights into the early stages of differentiation in these quail haplotypes (A and B) that can be expected to continue as divergence accumulates over the non‐recombining > 1200 genes included in the chromosome 1 inversion.

## Author Contributions

**Celia Vinagre‐Izquierdo:** data curation (lead), formal analysis (equal), investigation (lead), methodology (equal), software (lead), visualization (lead), writing – original draft (lead), writing – review and editing (equal). **Ines Sanchez‐Donoso:** conceptualization (equal), data curation (equal), formal analysis (equal), investigation (equal), methodology (equal), software (supporting), validation (lead), visualization (supporting), writing – review and editing (equal). **Jennifer A. Leonard:** investigation (supporting), methodology (equal), resources (equal), writing – review and editing (equal). **José Domingo Rodríguez‐Teijeiro:** conceptualization (equal), investigation (supporting), resources (supporting), writing – review and editing (equal). **Carles Vilà:** conceptualization (equal), funding acquisition (lead), investigation (equal), methodology (supporting), project administration (lead), resources (lead), supervision (lead), writing – original draft (equal), writing – review and editing (equal).

## Disclosure

*Benefit‐sharing statement*: Benefits from this research stem from the sharing of our data and results on publicly accessible databases.

## Conflicts of Interest

The authors declare no conflicts of interest.

## Supporting information


Data S1:

## Data Availability

Isotopic and morphological data, as well as scripts, are available as a ZIP file in the Supporting Information S1. GBS data are available in Dryad (pending accession number). Scripts for SNP Calling for Paired‐End GBS data and karyotype classification are also publicly available in a GitHub repository https://doi.org/10.5281/zenodo.14811512.

## Appendix A Linear Mixed Model Used to Study Differences in Isotopic Composition for Deuterium (δ^2^H), Carbon (δ^13^C) and Nitrogen (δ^15^N). ****p* < 0.001, ***p* < 0.01, and **p* < 0.05

**Table:**

Model H	m < − lme (H ~ karyotype*season, random = ~1|individual na.action = na.omit, data = data) Differences in δ^2^H due to karyotype: Chisq_2_ = 8.117, *p* = 0.017* season: Chisq_2_ = 8.007, *p* = 0.005** interaction of karyotype and season: Chisq_2_ = 1.818, *p* = 0.403
	Test (1) Effect of season by karyotype AA wintering vs. AA breeding: Sidak's estimate = 13.13, df = 29, *p* = 0.011* AB wintering vs. AB breeding: Sidak's estimate = 4.78, df = 29, *p* = 0.240 BB wintering vs. BB breeding: Sidak's estimate = 6.86, df = 29, *p* = 0.344
	Test (2) Effect of karyotype by season AA wintering vs. AB wintering: Sidak's estimate = −5.85, df = 29, *p* = 0.790 AA wintering vs. BB wintering: Sidak's estimate = −20.79, df = 29, *p* = 0.108 AB wintering vs. BB wintering: Sidak's estimate = −14.94, df = 29, *p* = 0.294 AA breeding vs. AB breeding: Sidak's estimate = −14.20, df = 29, *p* = 0.141 AA breeding vs. BB breeding: Sidak's estimate = −27.06, df = 29, *p* = 0.024* AB breeding vs. BB breeding: Sidak's estimate = −12.86, df = 29, *p* = 0.421
Model C	m < − lme (C ~ karyotype *season, random = ~1|individual, na.action = na.omit, data = data) Differences in δ^13^C due to karyotype: Chisq_2_ = 18.112, *p* < 0.0001*** season: Chisq_2_ = 28.523, *p* < 10^−8^*** interaction of karyotype and season: Chisq_2_ = 26.334, *p* < 10^−6^***
	Test (1) Effect of season by karyotype AA wintering vs. AA breeding: Sidak's estimate = 7.02, df = 29, *p* < 0.0001*** AB wintering vs. AB breeding: Sidak's estimate = 1.11, df = 29, *p* = 0.178 BB wintering vs. BB breeding: Sidak's estimate = 0.36, df = 29, *p* = 0.801
	Test (2) Effect of karyotype by season AA wintering vs. AB wintering: Sidak's estimate = 5.85, df = 29, *p* < 0.0001*** AA wintering vs. BB wintering: Sidak's estimate = 7.10, df = 29, *p* = 0.0001*** AB wintering vs. BB wintering: Sidak's estimate = 1.25, df = 29, *p* = 0.722 AA breeding vs. AB breeding: Sidak's estimate = −0.068, df = 29, *p* = 0.100 AA breeding vs. BB breeding: Sidak's estimate = 0.444, df = 29, *p* = 0.984 AB breeding vs. BB breeding: Sidak's estimate = 0.511, df = 29, *p* = 0.973
Model N	m < − lme (*N* ~ karyotype *season, random = ~1|individual na.action = na.omit, data = data) Differences in δ^15^N due to karyotype: Chisq_2_ = 3.274, *p* = 0.195 season: Chisq_2_ = 0.748, *p* = 0.387 interaction of karyotype and season: Chisq_2_ = 0.124, *p* = 0.940

## Appendix B Statistical Models Testing Morphological Differences Among Karyotypes AA, AB, and BB. ****p* < 0.001, ***p* < 0.01, and **p* < 0.05

**Table:**

Response variable	Explanatory variables, model statement and results	
	Karyotype	Sex
Weight (g)	m < − lm (weight ~ karyotype + sex, data = d)	
	*F*_2,28_ = 10.054, *p* < 0.001*** *Averaged over sex*: AA vs. AB Tukey estimate = −9.3, df = 28, *p* = 0.009** AA vs. BB Tukey estimate = −16.9, df = 28, *p* = 0.001** AB vs. BB Tukey estimate = −7.6, df = 28, *p* = 0.131	*F*_1,28_ = 8.001, *p* = 0.009**
Tarsus length (mm)	m < − lm (tarsus_length ~ karyotype + sex, data = d)	
	*F*_2,28_ = 10.266, *p* < 0.001*** *Averaged over sex*: AA vs. AB Tukey estimate = −1.2, df = 28, *p* = 0.003** AA vs. BB Tukey estimate = −1.8, df = 28, *p* = 0.001** AB vs. BB Tukey estimate = −0.6, df = 28, *p* = 0.356	*F*_1,28_ = 0.293, *p* = 0.593
Lipid band width (mm)	m < − lm (lipid_band_width ~ karyotype + sex, data = d)	
	*F*_2,28_ = 4.697, *p* < 0.017* *Averaged over sex*: AA vs. AB Tukey estimate = 2.3, df = 28, *p* = 0.034* AA vs. BB Tukey estimate = 3.1, df = 28, *p* = 0.044* AB vs. BB Tukey estimate = 0.7, df = 28, *p* = 0.796	*F*_1,28_ = 2.635, *p* = 0.116
Modified Holynski Index	m < − lm (modified_holynski_index ~ karyotype + sex, data = d)	
	*F*_2,28_ = 10.725, *p* < 0.001*** *Averaged over sex*: AA vs. AB Tukey estimate = 12.5, df = 28, *p* = 0.044* AA vs. BB Tukey estimate = 31.4, df = 28, *p* < 0.001*** AB vs. BB Tukey estimate = 18.8, df = 28, *p* = 0.019*	*F*_1,28_ = 3.400, *p* = 0.076
Cloacal aperture width (mm)	m < − lm (cloaca ~ karyotype + sex, data = d)	
	*F*_2,28_ = 5.409, *p* = 0.010* *Averaged over sex*: AA vs. AB Tukey estimate = −0.7, df = 28, *p* = 0.008** AA vs. BB Tukey estimate = −0.5, df = 28, *p* = 0.238 AB vs. BB Tukey estimate = 0.2, df = 28, *p* = 0.732	*F*_1,28_ = 11.970, *p* = 0.002**
Cheek pigmentation	fisher.test (karyotype_cheek)	fisher.test (sex_cheek)
	Fisher's exact test *p* = 0.037*, *N* = 32 *Only females*: Fisher's exact test *p* = 1, *N* = 14 *Only males*: Fisher's exact test *p* = 0.050*, *N* = 18	Fisher's exact test, *p* = 0.142, *N* = 32
Beak height (mm)	m < − lm (beak_height ~ karyotype + sex, data = d)	
	*F*_2,28_ = 1.251, *p* = 0.302	*F*_1,28_ = 2.473, *p* = 0.127
Beak width (mm)	m < − lm (beak_width ~ karyotype + sex, data = d)	
	*F*_2,28_ = 1.503, *p* = 0.240	*F*_1,28_ = 0.825, *p* = 0.372
Beak length (mm)	m < − lm (beak_length ~ karyotype + sex, data = d)	
	*F*_2,28_ = 5.504, *p* = 0.010* *Averaged over sex*: AA vs. AB Tukey estimate = −0.4, df = 28, *p* = 0.099 AA vs. BB Tukey estimate = −0.7, df = 28, *p* = 0.009** AB vs. BB Tukey estimate = −0.4, df = 28, *p* = 0.209	*F*_1,28_ = 0.440, *p* = 0.512
Beak pigmentation	fisher.test (karyotype_beakpigmentation)	fisher.test (sex_beakpigmentation)
	Fisher's exact test *p* = 0.013*, *N* = 32	Fisher's exact test, *p* = 0.113, *N* = 32
