# Supplementary figures and images for: Chromosomal Inversion Associated With Diet Differences in Common Quails Sharing Wintering Grounds
Source: Ecol Evol. 2025 Aug 20;15(8):e71792. doi: 10.1002/ece3.71792 (PMC12365396; doi:10.1002/ece3.71792)

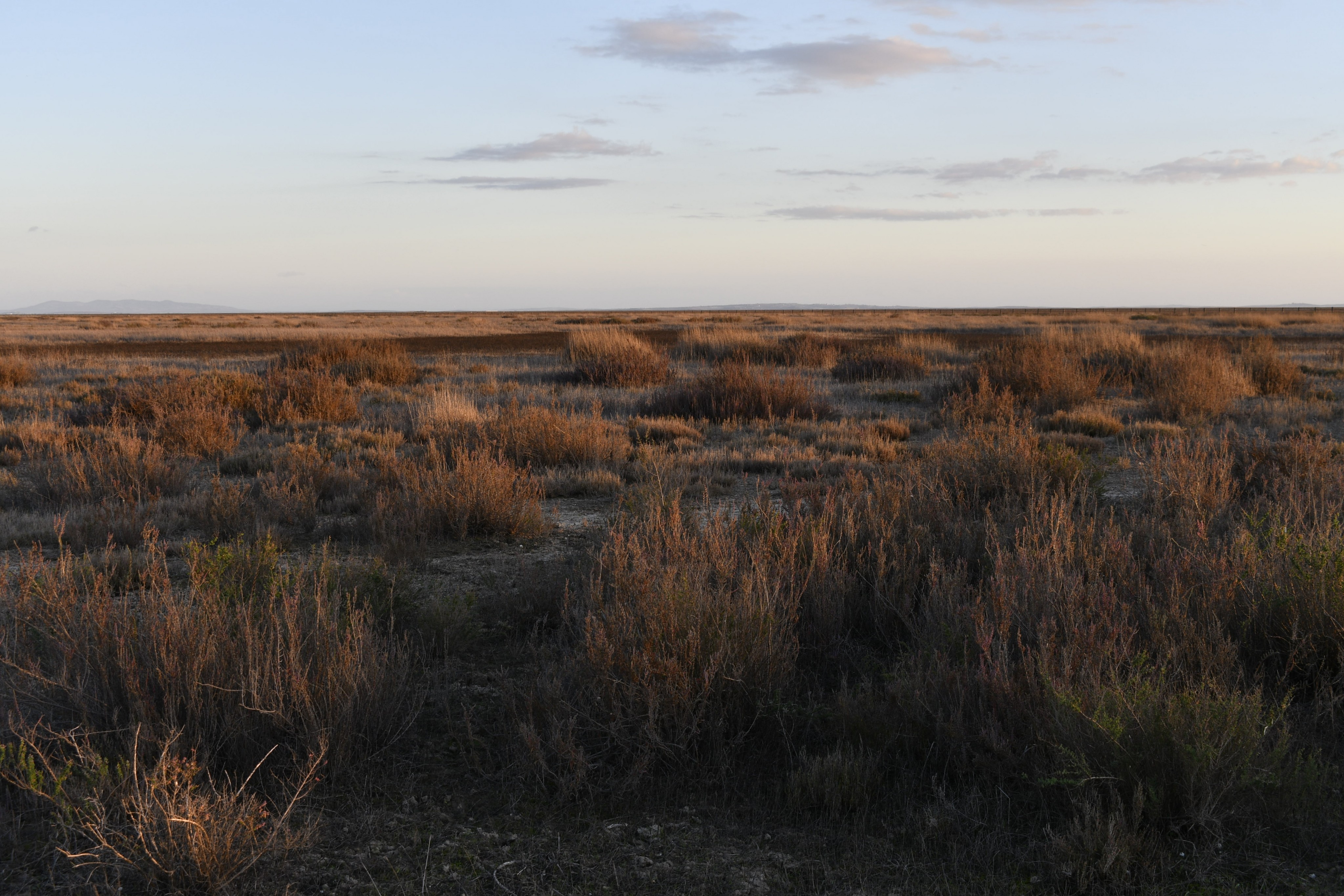

Supplement: Supplementary file 1 — Data S1: [file ECE3-15-e71792-s001.zip › Data-Analyses-Plots_Vinagre-Izquierdo et al_2025_MolEcol/1_Genomic_Analyses/4096-2731-max_bonita.jpg]

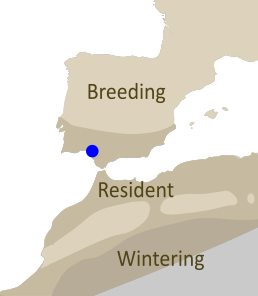

Supplement: Supplementary file 1 — Data S1: [file ECE3-15-e71792-s001.zip › Data-Analyses-Plots_Vinagre-Izquierdo et al_2025_MolEcol/1_Genomic_Analyses/mapa_invCaracoles.png]
